# Clinical characteristics, course, and outcomes of amyotrophic lateral sclerosis overlapping with pregnancy: a systematic review of 38 published cases

**DOI:** 10.1007/s10072-023-06994-4

**Published:** 2023-08-17

**Authors:** Abdullah Ashraf Hamad, Basma Ehab Amer, Aya Mustafa AL Mawla, Elarbi Goufa, Maya Magdy Abdelwahab, Ibrahim Serag

**Affiliations:** 1 Faculty of Medicine, Menoufia University, Shibin El-Kom, Menoufia Egypt; 2 Medical Research Group of Egypt, Negida Academy, Arlington, MA USA; 3 Faculty of Medicine, Benha University, Benha, Egypt; 4 Faculty of Medicine, Jordan University of Science and Technology, Irbid, Jordan; 5 Faculty of Medicine, University of Oran 1 - Ahmed Ben Bella, Oran, Algeria; 6 Faculty of Medicine, Helwan University, Cairo, Egypt; 7 Faculty of Medicine, Mansoura University, Mansoura, Egypt

**Keywords:** Amyotrophic lateral sclerosis, Motor neuron disease, ALS, Pregnancy, Case reports, Systematic review

## Abstract

**Objectives:**

Amyotrophic lateral sclerosis (ALS) is a rare and fatal neurodegenerative disease that can overlap with pregnancy, but little is known about its clinical characteristics, course, and outcomes in this context. This systematic review aimed to synthesize the current evidence on ALS overlapping with pregnancy.

**Methods:**

We comprehensively searched four databases on February 2, 2023, to identify case studies reporting cases of ALS overlapping with pregnancy. Joanna Brigs Institute tool was followed to assess the quality of the included studies.

**Results:**

Twenty-six articles reporting 38 cases were identified and included in our study. Out of the 38 cases, 18 were aged < 30 years. The onset of ALS was before pregnancy in 18 cases, during pregnancy in 16 cases, and directly after pregnancy in 4 cases. ALS progression course was rapid or severe in 55% of the cases during pregnancy, and this percentage reached 61% in cases with an onset of ALS before pregnancy. While ALS progression course after pregnancy was rapid or severe in 63% and stable in 37% of the cases. Most cases (95%) were able to complete the pregnancy and gave live birth. However, preterm delivery was common. For neonates, 86% were healthy without any complications.

**Conclusion:**

While pregnancy with ALS is likely to survive and result in giving birth to healthy infants, it could be associated with rapid or severe progression of ALS and result in a worse prognosis, highlighting the importance of close monitoring and counselling for patients and healthcare providers.

## Introduction

Amyotrophic lateral sclerosis (ALS), commonly known as Lou Gehrig’s disease, is a neurodegenerative disease that is rare, progressive, and ultimately fatal. It affects both the upper and lower motor neurons, leading to muscle weakness, atrophy, and paralysis over time [1]. Typically, ALS is diagnosed in middle age, with an average onset between 51 and 66 years, and is more prevalent in men than women [2]. Although ALS is uncommon in younger individuals, there have been isolated cases of ALS occurring during pregnancy [3, 4]. This overlap between ALS and pregnancy poses unique challenges, as the symptoms of ALS may worsen during pregnancy and complicate disease management. Additionally, the impact of ALS on the health of the developing fetus and pregnancy outcomes is not well understood.

The management of ALS involves a multidisciplinary approach that aims to manage symptoms, address complications, and provide supportive care to improve the quality of life of the patient [5]. This may involve a combination of medication, physical therapy, occupational therapy, speech therapy, and respiratory therapy [6]. Managing ALS during pregnancy can be particularly challenging, as pregnant women with ALS may experience worsening of symptoms or complications associated with the disease. For example, respiratory function may be compromised due to the increased metabolic demands of pregnancy, and the use of certain medications may be contraindicated during pregnancy [7]. Therefore, close monitoring and coordinated care between the patient’s healthcare providers are essential to manage the disease effectively during pregnancy and ensure the best possible outcomes for both the mother and the baby.

Due to the rarity and complexity of ALS during pregnancy, there is a need to enhance our understanding of the disease and its management in this context. This article aimed to investigate the relationship between ALS and pregnancy, the course of ALS during pregnancy, and the potential impact of ALS on pregnancy outcomes.

## Methods

PRISMA (Preferred Reporting Items for Systematic Reviews and Meta-analyses) guidelines were followed during presenting this review [8].

### Search strategy and inclusion criteria

On February 2, 2023, a comprehensive search was conducted on PubMed, EBSCO, Scopus, and Web of Science using the following search terms: (“Motor Neuron Disease” OR “Motor System Disease” OR “Gehrig Disease” OR “Charcot Disease” OR “Amyotrophic Lateral Sclerosis” OR “Guam Disease” OR “ALS”) AND (“pregnant” OR “pregnancy”). No search restrictions or filters were applied. Studies had to meet the following criteria to be included in our review: (a) be case reports or case series describing cases of ALS overlapping with pregnancy; (b) be published after 1980 to avoid outdated cases; and (c) be reported in English.

### Outcomes of interest

The primary outcomes of interest in this review included the course of ALS during and after pregnancy, as well as the pregnancy and neonatal outcomes of women with ALS. The progression of ALS was assessed based on the rate of disease progression during pregnancy and after delivery, as reported in the included studies. The course of the disease was determined based on the reported changes in the patient’s functional status, clinical features, and survival during and after pregnancy. The progression of ALS was classified as stable if there was no significant change in these measures or as rapid or severe progression if there was a significant decline, while the pregnancy and neonatal outcomes of interest included gestational age at delivery, mode of delivery, neonatal birth weight and health status, and the incidence of neonatal morbidity.

### Screening and data extraction

Without removing duplicates from the identified records [9], titles and abstracts were independently screened against the inclusion criteria by two authors. Then, the full texts of articles identified from that stage were retrieved and screened by a third author to make the final decision. After identifying the included articles, two authors extracted the data independently with the help of an online data form. The extracted data included: (a) study year and country; (b) age and gestational history; (c) onset of ALS; (d) course of ALS during and after pregnancy; (e) pregnancy and neonatal outcomes; and (f) quality assessment domains. Any discrepancies in screening or data extraction were resolved through discussion with a third author.

### Quality assessment

The quality assessment of the included studies was performed using the Joanna Briggs Institute (JBI) tool for case reports [10]. Two authors independently assessed the quality of each study, and any discrepancies were resolved through discussion with a third author. The JBI tool includes specific criteria for evaluating the methodological quality of case reports and case series [10].

## Results

### Characteristics of the included studies

**Fig. 1:**
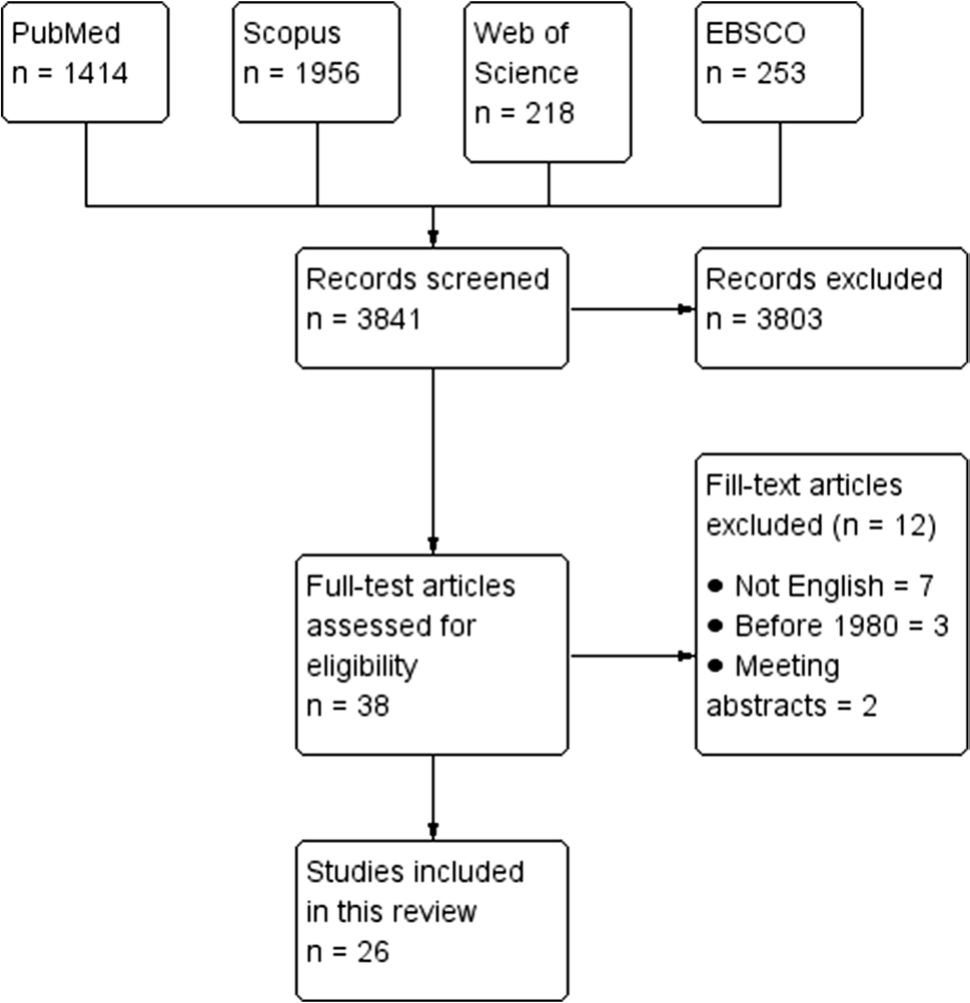
The PRISMA flow diagram

Following the search strategy, we identified 3841 articles. After applying our inclusion criteria, we identified 26 articles reporting 38 cases of ALS overlapping with pregnancy, with 18 cases occurring in women under 30 years of age [3, 4, 7, 11–33]. Figure 1 summarizes the selection process of the included studies. The onset of ALS was before pregnancy in 18 cases, during pregnancy in 16 cases, and directly after pregnancy in 4 cases. Out of 21 cases with reported obstetric history, 7 cases were primigravida. Table 1 summarizes the demographic characteristics, clinical course, and outcomes of the included studies. The quality assessment of the included studies is summarized in Table 2.

**Table 1:** The demographic characteristics, clinical course, and outcomes of the included cases

Study ID	Country	Age	Obstetric history	Family history of ALS	Onset of ALS	ALS during pregnancy	Pregnancy outcome	Neonatal outcome	ALS after pregnancy
Martinez 2014 [27]	Mexico	37	Primigravida	None	At the 9th weeks of gestation with upper left limb weakness	Stable progression	Gave a live birth at the 38th week by cesarean section	Healthy boy weighting 3200 g and remained healthy to the end of follow-up	Progressed severely until she had gastrostomy and ventilatory support
Masrori 2022 [28]	Belgium	34	NR	NR	At the 6th months of gestation with respiratory symptoms	NR	Gave a live birth by vaginal delivery	Healthy neonate	Progressed severely till she became permanently ventilator-dependent and died 20 months after the disease onset
Miranda 2014 [29]	Colombia	37	Gravida 3, Para 2	NR	Three years before the current pregnancy	Progressed severely showing marked dysarthria, muscular atrophy, and tetraplegia with hyperreflexia; respiratory failure was diagnosed.	Gave a live birth at the 32nd week by vaginal delivery	Female neonate weighing 1190 g; she was admitted to the neonatal ICU because of low birth weight but was discharged home a week later.	Showed stable progression; she was discharged home 1 month after delivery and continued mechanical ventilatory care.
Pathiraja 2020 [30]	Sri Lanka	32	Gravida 5, Para 2	NR	Two years before the current pregnancy	Progressed rapidly and showed a severe restrictive type lung disease	Gave a live birth at the 34th week of gestation by cesarean section	Healthy neonate weighing 2500 g	NR
Porto 2018 [3]	Canada	29	Primigravida	None	Four and half years before the current pregnancy	Worsened rapidly with severe lower limb symptoms and dysphagia	Gave a live birth at 37 + 4 weeks by cesarean section	Healthy neonate weighing 3022 g	Progressed rapidly 6 weeks after delivery with severe weakness, dysphagia, and dysarthria
Sarafov 2009 [4]	Germany	28	Primigravida	None	One year before the current pregnancy with weakness of the right upper extremity	Stable progression	Gave a live birth at the 40th week of gestation by vaginal delivery	Healthy neonate with healthy grow till the end of follow-up	Symptoms were stable then started getting worse after 8 months of the delivery. She became bedridden 3 years after the onset.
		30	Gravida 2, Para 1	None	Two years before the current pregnancy	Rapid progression during pregnancy showing progressive dysphagia and cachexia	Gave a live birth at the 34th week of gestation by cesarean section	Female neonate weighing 2000 g with intrapartal asphyxia; she developed well since then.	Progressed with severe symptoms; the patient was put on long-term invasive mechanical ventilation and died 11 months after the delivery.
Scalco 2012 [31]	Brazil	28	NR	NR	Nine months before the current pregnancy	Stable progression	Gave a live birth at the 37th of pregnancy	Male neonate weighing 2260 g showed a small atrial communication and a small patent ductus arteriosus.	NR
Sobrino-Bonilla 2004 [32]	USA	32	Gravida 2, Para 1	NR	One year before the current pregnancy	Rapid progression during pregnancy	Gave a live birth at 38 + 6 weeks of gestation by vaginal delivery	Male healthy neonate	Stable progression
Solodovnikova 2021 [33]	Ukraine	25	NR	None	At the 6th month of the current pregnancy	Rapid progression showing dyspnea, weakness, and difficulties walking	Gave a live birth at the 38th week of pregnancy by cesarean section	Healthy neonate	Progressed with severe symptoms; she died 6 months after the onset of ALS with respiratory failure.
Vincent 1995 [11]	Uruguay	27	Para 1	NR	Two months before the current pregnancy	Progressed severely with severe dysphonia and dysphagia	Gave a live birth at the 32nd week of gestation	Healthy male neonate weighing 2800 g	Progressed severely showing a restrictive ventilatory insufficiency; her condition improved after that.
Wang 2021 [12]	China	34	Gravida 4, Para 3	None	At the third month of pregnancy with mild dysphonia and stiffness of gait	Progressed rapidly with hyperreflexia at the lower limbs and denervation in active muscle	Gave a live birth at the 36th week of gestation	Healthy male neonate weighing 2720 g	Stable profession
Weiss 2006 [13]	USA	21	Gravida 2, Para 1	None	At the first weeks of pregnancy with progressive left arm weakness and muscle wasting	Stable progression	Gave a live birth at the 34th week of gestation by caesarean section	Healthy female neonate weighing 2154 g	Progressed severely after delivery with worsened vital capacity, weakness, and atrophy
Xiao 2017 [14]	China	27	Gravida 2, Para 0	NR	One year before the current pregnancy	Progressed rapidly with weakness, spasticity, and restrictive pulmonary dysfunction	Gave a live birth at the 35th week of gestation	Healthy female neonate weighing 2550 g by cesarean section	NR
Ali 2022 [15]	UK	25	NR	None	Three years before the current pregnancy	Stable progression	Gave a live birth at 34 + 4 weeks gestation	Healthy male neonate weighing 2760 g	Stable progression
Tyagi 2001 [16]	Ireland	29	NR	None	At the 6th month of the current pregnancy with ataxia, lower limb weakness, and dysarthria	Stable progression	Gave a live birth	Healthy male neonate	Stable progression
Bazán-Rodríguez 2022 [7]	Mexico	28	NR	NR	Two months before the current pregnancy with bulbar symptoms and drop head	Rapid progression to mechanical ventilation	Gave a live birth at 30 + 4 weeks of gestation	Healthy neonate	Progressed severely to invasive ventilation and gastrostomy
Chiò 2003 [23]	Italy	27	Primigravida	NR	Started at the 6th month of pregnancy with upper limp weakness	Stable progression	Gave a live birth at 40 weeks by vaginal delivery	Healthy female neonate in good health to the end of follow-up	Progressed rapidly to severe dysarthria and dysphagia 6 months after the delivery and underwent percutaneous endoscopic gastrostomy
		29	Primigravida	NR	Started at the 5th month of pregnancy with lower limb atrophy and weakness	Stable progression	Gave a live birth at 39 weeks by vaginal delivery	Healthy male neonate	Stable progression
		33	Gravida 2	NR	Started at the 3rd month of pregnancy with shoulder girdle	Progressed severely with respiratory impairment	Interrupted pregnancy in the 5th month	–	The patient died 3 months later, due to respiratory failure.
		38	Gravida 3	NR	Started 1 year before the current pregnancy with upper limb weakness and atrophy	Stable progression	Gave a live birth at 34 weeks	Healthy male infant	Stable progression
Hancevic 2019 [24]	Croatia	41	NR	None	One year before the current pregnancy	Stable progression	Gave a live full-term birth	NR	She died 52 months from the onset of symptoms.
		35	NR	None	At the beginning of the current pregnancy	Stable progression	Gave stillbirth in the 23rd week of pregnancy	–	NR
		32	NR	None	Two years before the current pregnancy	Stable progression	Gave a live full-term birth by vaginal delivery	Healthy male neonate	Rapidly progressed reaching ALSFRS-R 14 within 6 months
Hilton 2002 [17]	UK	25	NR	NR	In the last month of pregnancy	NR	Gave a live birth by a caesarian section	NR	Progressed rapidly with weakness in respiratory muscles; she was ventilated for 27 months prior to death.
Jacka 1998 [18]	Canada	31	Primigravida	yes	In the 8th week of gestation with progressive leg weakness	Rapid progression to tetraparesis and respiratory insufficiency	Gave a live birth by cesarean section	Healthy female neonate 2810 g	Progressed severely to respiratory failure but improved after long-term ventilation, tracheostomy, and gastrostomy
Kawamichi 2010 [19]	Japan	34	Primigravida	None	Four years before the current gestation	Stable progression	Gave a live birth at 38 weeks of gestation by vaginal delivery	Healthy female neonate weighing 2280 g	Stable progression
Kock-Cordeiro 2018 [20]	Netherlands	25	Gravida 2, Para 1	NR	At the 27th week of pregnancy	Rapid progression with respiratory symptoms	Gave a live birth by cesarean section	Male neonate admitted to ICU with grade II respiratory distress syndrome.	NR
Leveck 2005 [21]	Canada	25	Para 2	NR	At the 22nd week of gestation	Rapid progression with severe dysphagia and respiratory impairment	Gave a live birth at 34.5 weeks	Healthy female infant weighing 2305 g	Progressed severely; she died at 9-month postpartum.
Lunettaa 2014 [25]	Italy	29	NR	NR	During labor	NR	Gave a live birth	Healthy neonate	Progressed severely with invasive ventilation (still alive 110 months after delivery)
		32	NR	NR	At the 24th week of the pregnancy	NR	Gave a live birth	Healthy neonate	Rapid progression (deceased 37 months after delivery)
		38	NR	NR	One week after the delivery	NR	Gave a live birth	Healthy neonate	Slow deterioration (still alive 51 months after delivery)
		42	NR	NR	One month from the delivery	NR	Gave a live birth	Healthy neonate	Severe progression with percutaneous endoscopic gastrostomy three (deceased 49 months after delivery)
		32	NR	NR	One month from the delivery	NR	Gave a live birth	Healthy neonate	NR
Lupo 1993 [26]	USA	28	Para 2–0-0–2	Yes	At 36 weeks of gestation	Stable progression	NR	NR	Stable progression
		29	NR	Yes	Nine months before pregnancy	Progressed rapidly with respiratory insufficiency	Gave a live birth at 34 weeks by cesarean section	Two healthy male infants weighing 2270 g	Died due to respiratory failure 6 weeks after delivery
		34	Para 4–0-0–4	NR	Two years before gestation	Progressed rapidly to full-time ventilator support	Gave a live birth at the 33rd week by vaginal delivery	Healthy neonate weighing 2330 g	Stable progression; her status was unchanged 1.5 year after delivery.
Marsli 2020 [22]	Morocco	34	NR	None	One year before pregnancy	Progressed rapidly showing diffused fasciculation, followed by weakness affecting all four limbs	Gave a live birth	Healthy infant	Progressed severely towards respiratory dysfunction and non-invasive ventilation was required;. the patient died 9 months after the disease onset.

*ALS*, amyotrophic lateral sclerosis; *NR*, not reported; *ALSFRS-R*, Revised Amyotrophic Lateral Sclerosis Functional Rating Scale

**Table 2:** The quality assessment of the included studies using the JBI tool for case reports

Study ID	Were the patient's demographic characteristics clearly described?	Was the patient's history clearly described and presented as a timeline?	Was the current clinical condition of the patient on presentation clearly described?	Were diagnostic tests or assessment methods and the results clearly described?	Was the intervention (s) or treatment procedure (s) clearly described?	Was the post-intervention clinical condition clearly described?	Were adverse events (harms) or unanticipated events identified and described?	Does the case report provide takeaway lessons?
Martinez 2014 [27]	Y	Not clear	Y	Y	Y	Y	Y	Y
Masrori 2022 [28]	Y	Not clear	Y	N	Y	NA	NA	Y
Miranda 2014 [29]	Y	Not clear	Y	N	Y	Y	Not clear	Y
Pathiraja 2020 [30]	Y	Not clear	Y	N	NA	NA	NA	Y
Porto 2018 [3]	Y	Y	Y	Not clear	NA	NA	NA	Y
Sarafov 2009 [4]	Y	Y	Y	Y	NA	NA	NA	Y
Scalco 2012 [31]	Y	Not clear	Y	Y	Y	Y	Y	Y
Sobrino-Bonilla 2004 [32]	Y	Y	Y	N	NA	NA	NA	Y
Solodovnikova 2021 [33]	Y	Y	Y	Y	NA	NA	NA	Y
Vincent 1995 [11]	Y	Y	Y	Y	NA	NA	NA	Y
Wang 2021 [12]	Y	Not clear	Y	Y	Y	Y	Y	Y
Weiss 2006 [13]	Y	Not clear	Y	N	NA	NA	NA	Y
Xiao 2017 [14]	Y	Y	Y	Y	Y	Y	Y	Y
Ali 2022 [15]	Y	Y	Y	N	NA	NA	NA	Y
Tyagi 2001 [16]	Y	Not clear	Y	N	NA	NA	NA	Y
Bazán-Rodríguez 2022 [7]	Y	Not clear	Y	Y	NA	NA	NA	Y
Chiò 2003 [23]	Y	Y	Y	N	NA	NA	NA	Y
Hancevic 2019 [24]	Y	Y	Not clear	Y	NA	NA	NA	Y
Hilton 2002 [17]	Y	Y	Not clear	N	NA	NA	NA	Y
Jacka 1998 [18]	Y	Y	Y	N	NA	NA	NA	Y
Kawamichi 2010 [19]	Y	Y	Y	Y	Y	Y	Y	Y
Kock-Cordeiro 2018 [20]	Y	Y	Y	Y	Y	Y	Y	Y
Leveck 2005 [21]	Y	Y	Y	N	NA	NA	NA	Y
Lunettaa 2014 [25]	Y	Y	Not clear	N	NA	NA	NA	Y
Lupo 1993 [26]	Y	Y	Y	N	NA	NA	NA	Y
Marsli 2019 [22]	Y	Y	Not clear	N	NA	NA	NA	Not clear

*JBI*, Joanna Briggs Institute; *Y*, yes; *N*, no; *NA*, not applicable

### ALS course

The progression of ALS was rapid or severe in the majority of cases during pregnancy, with 17 out of 31 cases (55%) experiencing rapid or severe progression and 45% experiencing stable progression. Among cases with an onset of ALS before pregnancy, 11 out of 18 cases (61%) showed rapid or severe progression during pregnancy, while among cases with an onset of ALS during pregnancy, 6 out of 13 cases (46%) showed rapid or severe progression during pregnancy. After pregnancy, 20 out of 32 cases (63%) showed rapid or severe progression, and 37% showed stable progression of ALS. The clinical course of ALS for each case is summarized in Table 1.

### Pregnancy and neonatal outcomes

Out of 37 cases, 35 (95%) were able to complete their pregnancies and give live birth, while the remaining two cases resulted in stillbirths [23, 24]. However, only 33% (8 out of 24) of these completed pregnancies reached 38 weeks. The delivery method was cesarean in 10 cases, vaginal in 9 cases, and not reported in the other cases (Table 1). For neonatal outcomes, most cases (25 out of 29) resulted in normal healthy infants without complications. In the other four cases, three infants showed transient complications after delivery [4, 20, 29], while one infant had small atrial communication and a small patent ductus arteriosus [31].

## Discussion

### Main findings

The aim of this systematic review was to investigate the impact of pregnancy on the progression of ALS and to examine neonatal and pregnancy outcomes in affected patients. Our results support the hypothesis that pregnancy increases the potential for severe and rapid ALS progression, regardless of the timing of ALS onset in relation to pregnancy. Also, the majority of cases reviewed demonstrated that ALS had no negative impact on neonatal or pregnancy outcomes.

### Interpretations

ALS is a neurodegenerative disorder which affects the motor ganglia of both upper and lower motor neurons [1]. It is more common in men, and its incidence is increased after the fifth decade [2, 34]. Therefore, it rarely affects women in their child bearing periods. This can explain why ALS overlapping with pregnancy is very rare. To date, the exact etiology of ALS progression is still unknown; however, sex-related factors, such as hormones, may play a role in its progression. Previous animal studies showed that both estrogen and progesterone are protective against ALS progression in mice models [35]. The abrupt decrease in the levels of these hormones after delivery may explain the rapid or severe progression of ALS after pregnancy. However, this theory cannot explain the rapid progression of ALS during pregnancy when these hormones are normally elevated. In addition, a recent case control study showed that the odds of ALS were decreased in women receiving estrogen-progesterone hormone replacement therapy in the Netherlands; however, this protective effect was not observed in women in both Italy and Ireland [36]. Similarly, this protective effect could not be observed in earlier studies which enrolled postmenopausal women receiving estrogen replacement therapy [37, 38]. The conflicting results of these studies suggest that hormonal changes are not the only factor which contributes to ALS pathogenesis and progression. Genetic factors, such as mutations in vascular endothelial growth factor premotor and superoxide dismutase genes, may also contribute to the pathogenesis of ALS [25, 39]. Moreover, the inflammatory changes during pregnancy increase the oxidative stress, which further induces these genetic mutations increasing ALS susceptibility [30].

ALS primarily affects the voluntary muscles causing their progressive atrophy; however, it may affect the respiratory system which is mainly involuntary [32]. This can be attributed to the voluntary component of the diaphragm and costal muscles which can further be affected by ALS. In pregnant women, the enlarging uterus leads to upward elevation of the diaphragm and increases the risk of diaphragmatic fatigue [40]. Moreover, pregnancy, delivery, and the immediate postpartum period require an increase in respiratory work, which is normally achieved through diaphragmatic breathing [30]. Hence, respiratory muscle affection is the main concern in ALS overlapping with pregnancy [30]. The rapid deterioration of ALS progression after delivery, regardless of its onset in relation to pregnancy, may be attributed to the failure to meet the increased demand of respiratory work required during pregnancy, delivery, and the postpartum period [14]. Therefore, vaginal delivery, which requires more respiratory effort than cesarean section, is preferred only when the patients’ respiratory condition is stable. Otherwise, cesarean section is considered a safer option [14].

### Strengths and limitations

To the best of our knowledge, our study is the first systematic review to gather the evidence from all published case studies regarding ALS overlapping with pregnancy. All included reports were peer-reviewed case studies. In addition, we excluded studies published before 1980 to avoid outdated cases. Finally, we did not only explore the impact of pregnancy on ALS progression but also explored pregnancy and neonatal outcomes in ALS with pregnancy. Nevertheless, our study is not free of limitations. Firstly, the course of ALS was primarily determined based on clinical and functional characteristics reported in the cases, rather than on objective or quantitative measures such as the Revised Amyotrophic Lateral Sclerosis Functional Rating Scale, which were rarely reported. Secondly, the lack of genetic information in our analysis may have limited our ability to explain the progressive course of ALS, as genetic mutations may explain the ALS progressive course especially in young age [41, 42]. We also observed heterogeneity among the included studies in terms of ALS onset in relation to pregnancy and phenotypic characterization of the patients. This may have also limited the generalizability and validity of the findings. Lastly, we excluded non-English studies, which may potentially reduce the comprehensiveness of our systematic review. Together, all these limitations restrict the generalizability of our findings.

### Clinical implications and recommendations

Our results suggest that respiratory support is essential to meet the increased demands during pregnancy, delivery, and even the postpartum period in ALS-affected women. This may reduce the potential risk for respiratory failure and slow ALS progression after delivery. Moreover, monitoring of respiratory functions in pregnant females with ALS is crucial, particularly during the third trimester to determine the best option for delivery. One included study reported the use of riluzole during pregnancy, which was associated with neonatal cardiac malformations [31]; however, another study showed that riluzole did not cause any maternal or fetal side effects [19]. Further studies are required to truly explore the impact of maternal use of riluzole during pregnancy on both maternal and neonatal outcomes.

## Conclusion

While pregnancy with ALS is likely to survive and result in giving birth to healthy infants, it could be associated with rapid or severe progression of ALS and result in a worse prognosis, highlighting the importance of close monitoring and counselling for patients and healthcare providers. Further research is necessary to gain a better understanding of the pathophysiology and optimal management of ALS in pregnancy. Future studies should consider including genetic assessments and ALS functional scores to enhance our understanding of ALS in this context. Overall, a better understanding of ALS in pregnancy can help guide clinical decision-making and improve outcomes for both the mother and infant.

## Data Availability

Not applicable.
